# Pericardiocentesis Indications and Complications: A Retrospective Observational Study in a Tertiary Care Hospital in Karachi, Pakistan

**DOI:** 10.7759/cureus.10102

**Published:** 2020-08-28

**Authors:** Abdul Baqi, Intisar Ahmed

**Affiliations:** 1 Cardiology, Aga Khan University Hospital, Karachi, PAK; 2 Cardiology, Aga Khan University Hospital, Karachi, PAK

**Keywords:** cardiac tamponade, pericardiocentesis, echocardiography, fluoroscopy, pericardial effusion

## Abstract

Introduction: Pericardiocentesis is crucial for the diagnosis and management of diseases responsible for significant pericardial effusions. Pericardiocentesis was performed the first time by Riolanus for cardiac tamponade. He described the process of trephination of the sternum to remove the abnormally accumulated fluid from the pericardial space. However, with the advancement of expertise in echocardiography assisted procedures, echocardiography-guided diagnostic and therapeutic pericardiocentesis is now considered standard clinical practice in the treatment of pericardial effusions.

Objectives: We aim to study different causes of pericardial effusion and indications of pericardiocentesis as well as complications associated with it in our population.

Methods: This is a retrospective observational study done at Aga Khan University Hospital, Karachi. We reviewed hospital record files of 66 patients admitted to Aga Khan University Hospital from January 2010 to December 2019 who underwent pericardiocentesis.

Results: Out of 66 patients, 43 (65.2%) were male. The mean age of the study population was 48.59±18.9 years and 41 (62.1%) of them had underlying active malignancy with hematological malignancies being most common followed by lung carcinoma. In the majority of patients (71.2%), pericardiocentesis was performed at the bedside, and the rest of them (28.8%) underwent pericardiocentesis in the cardiac catheterization laboratory. Of all the patients, 46 (69.7%) underwent pericardiocentesis under echocardiography guidance and 18 (27.3%) required fluoroscopy. Successful pericardiocentesis was performed in 65 (98.5%) of the patients, two (3%) patients developed access site infection and only one (1.5%) patient became hemodynamically unstable while undergoing pericardiocentesis

Conclusion: Malignancy, predominantly lymphoma, is the most common cause of pericardial effusion requiring pericardiocentesis. Dyspnea is the most common symptom of presentation with cardiac tamponade. Echocardiography is the commonly used imaging modality for pericardiocentesis. Bedside setting is the most common setting used for pericardiocentesis. Imaging guided pericardiocentesis has a very high success and low complication rate.

## Introduction

Pericardiocentesis is crucial for the diagnosis and management of diseases responsible for significant pericardial effusions. Pericardiocentesis was performed the first time by Riolanus for cardiac tamponade. He described the process of trephination of the sternum to remove the abnormally accumulated fluid from the pericardial space [1]. Blind pericardiocentesis without any imaging assistance was used in the past, but that failed to gain wide support due to high morbidity, life-threatening complications more than 20%, and mortality approaching 6% [2].

However, with the advancement of expertise in echocardiography assisted procedures in the 1970s, pericardiocentesis became crucial for the diagnosis and management of diseases responsible for significant pericardial effusions. Echocardiography-guided pericardiocentesis both diagnostic and therapeutic at this time is considered the ideal clinical practice for the management of pericardial effusions [3].

Pericardiocentesis can be done without harm in hemodynamically stable patients in an outpatient setting. Pericardiocentesis can be performed quickly by an expert cardiologist in hemodynamically unstable patients to relieve symptoms of cardiac tamponade [4]. In medium to large-sized pericardial effusions, echocardiography-assisted pericardiocentesis has high success rates of >95% with very little risk. The morbidity rate is approximately 1-3% and the mortality rate associated directly with the procedure is less than 1% [5].

In 2005, Khan et al. described the clinical and echocardiographic features of Pakistani patients with pericardial effusion requiring pericardiocentesis and reported a success rate of around 93% for echocardiographic pericardiocentesis [6]. Since then, there is no data on procedural success and complications of pericardiocentesis in our population. We aim to study the causes of pericardial effusion, indications of pericardiocentesis in our population as well as complications associated with it, as this will help us understand procedural success and complications of the procedure in the contemporary era of advanced techniques.

## Materials and methods

Study population

This is a retrospective observational study done at Aga Khan University Hospital, Karachi, which is a multidisciplinary tertiary care hospital with a cardiology and cardiac surgery center that is one of the main referral health facilities in Pakistan.

This study was approved by Aga Khan University ethical review committee (ERC No. 2019-2253-5880). We reviewed hospital record files of consecutive 66 patients, admitted to Aga Khan University Hospital from January 2010 to December 2019, who underwent pericardiocentesis.

For all patients, demographic information including age, gender, and symptoms on presentation was recorded after reviewing written medical records. Comorbidities were tabulated including diabetes mellitus, hypertension, coronary artery disease, congestive heart failure, cardiomyopathy, and valvular heart disease. Echocardiographic data were reviewed for the size of pericardial effusion, distribution of pericardial effusion, echocardiographic signs of tamponade, and left ventricular systolic function. The size of the pericardial effusion was graded as small (<10 mm), medium (10-20 mm), and large (20 mm) on bases of largest echo-free space, when viewed from the transthoracic echocardiographic window. Etiology of pericardial effusion was established based on clinical reasoning, lab reports, echocardiography report, and the results of the pericardial fluid analysis. 

Procedure

After informed consent, pericardiocentesis was performed by a trained cardiologist under standard aseptic measures. Details of procedure including access site, anesthesia, imaging modality, and complications were recorded. Procedure success was defined as successful drainage pericardial fluids with the placement of a pericardial drain if required.

In-hospital monitoring

All patients were followed from the time of the procedure to the time of discharge or death. Hemodynamic parameters including heart rate and blood pressure were recorded at the time of procedure and post-procedure till the time of discharge. Complications related to pericardiocentesis were recorded.

Statistical analysis

All statistical analyses were performed using the Statistical Package for Social Sciences (SPSS) version 23 (IBM Corp., Armonk, NY). Continuous variables were expressed as mean value ± standard deviation and categorical variables were expressed as frequencies and percentages.

## Results

Baseline characteristics

Out of 66 patients, 43 (65.2%) were male, and the mean age of the study population was 48.59±18.9 years. Hypertension and diabetes mellitus was found in 36 (54.5%) and 25 (27.2%) patients, respectively. Coronary artery disease, chronic kidney disease, and chronic obstructive pulmonary disease were found in four (6.1%), six (9.1%), and three (4.5%) of the patients, respectively. Out of 66 patients, 41 (62.1%) of them had underlying active malignancy with hematological malignancies being most common followed by lung carcinoma (Table 1).

**Table 1:** Patients demographics and baseline characteristics (n = 66) COPD: chronic obstructive pulmonary disease

Variable	No. (%)
Age, mean (SD) year	48.59±18.9
Sex	
Male	43 (65.2)
Female	23(34.8)
Active cancer	41(62.1)
Hypertension	36 (54.5)
Diabetes Mellitus	25(27.2)
Ischemic heart disease	4(6.1)
Congestive heart failure	7(10.6)
Chronic kidney disease	6(9.1)
COPD	3(4.5)

Presenting symptoms and echocardiographic features

The majority of patients (63, 95.5%) presented with shortness of breath, and only three of them presented with hypotension (Table 2). Echocardiogram revealed large pericardial effusion in 61 (92.6%) patients, medium-sized effusion in five (7.4%) patients and all of them had echocardiographic features of tamponade including early diastolic right atrial and end-diastolic right ventricular collapse, abnormal respiratory variation in tricuspid and mitral flow velocities, dilated inferior vena cava with lack of inspiratory collapse. The mean left ventricular ejection fraction of the study population was 53.18±7.7% (Table 3).

**Table 2:** Presenting symptoms.

Symptoms	No (%)
Dyspnea	63(95.5)
Hypotension	3(4.5)

**Table 3:** Echocardiographic characteristics.

Variable	No (%)
Left ventricular ejection fraction mean (SD)	53.18+7.72
Size of pericardial effusion	
Large	61(92.4)
Medium	5(7.6)
Tamponade physiology	
Yes	66(100)
No	0
Distribution of pericardial effusion	
Circumferential	66(100)
Loculated	0(0)

Cause of pericardial effusion and indication of pericardiocentesis

In 41 (62.1%) patients, malignancy was the cause of pericardial effusion followed by viral pericarditis, tuberculous pericarditis, and uremic pericarditis in four (6.1%) patients each. Connective tissue disorders, hypothyroidism, and right heart failure were the cause of pericardial effusion in three (4.5%), two (3.0%), and two (3.0%) patients, respectively. One (1.5%) patient developed pericardial effusion due to post-myocardial infarction pericarditis (Table 4). Half of the patients underwent pericardiocentesis for therapeutic and the rest of them underwent diagnostic as well as therapeutic purposes.

**Table 4:** Etiology of cardiac tamponade. CABG: coronary artery bypass grafting

Etiology	No (%)
Active cancer	41(62.1)
Lung	12(18.2)
Leukemia/lymphoma	16(24.2)
Breast	3(4.5)
Gastrointestinal	2(3.0)
Head and neck	2(2.0)
Thymoma	2(3.0)
Thyroid gland	1(1.5)
Gynecological	1(1.5)
Genitourinary	1(1.5)
Viral pericarditis	4(6.1)
Tuberculous pericarditis	4(6.1)
Uremic pericarditis	4(6.1)
Post MI pericarditis	1(1.5)
Connective tissue disorders	3(4.5)
Hypothyroidism	2(3.0)
Right heart failure	2(3.0)
Post CABG	1(1.5)
Others	4(6.1)

Procedure and procedure-related complications

In the majority of patients (47, 71.2%), pericardiocentesis was performed at the bedside and in the rest (19, 28.8%) pericardiocentesis was performed at the cardiac catheterization laboratory. Of all the patients, 46 (69.7%) underwent pericardiocentesis under echocardiography guidance and 18 (27.3%) required echocardiography for localization and fluoroscopy for confirmation of effusion. Two of the patients underwent the blind procedure without imaging guidance due to the life-threatening condition of the patient and the non-availability of echocardiography machine at the bedside during the procedure (Table 5).

**Table 5:** Procedural details.

Variable	No (%)
Setting of Pericardiocentesis	
Bedside	47(71.2)
Cath lab	19(28.8)
Conscious sedation	65(98.5)
General anesthesia	1(1.5)
Guided	
Echo	46(69.7)
Echo and Fluoroscopy	18(27.3)
Blind without imaging	2(3.0)
Site of entry	
Subcostal	66(100)
Apical	0(0)
Catheter used	
Pigtail	61(92.4)
Sheath	5(7.6)

In the majority of patients, pericardiocentesis was performed under local anesthesia via a subcostal approach and a pigtail catheter was inserted in pericardial space. Successful pericardiocentesis was performed in 65 (98.5%) of the patients, two (3%) patients developed access site infection and only one (1.5%) patient became hemodynamically unstable while undergoing pericardiocentesis due to relative hypovolemia associated with underlying infection (Table 6).

**Table 6:** Complications of pericardiocentesis.

variables	No (%)
Infection	2(3.0)
Hemodynamic derangement	1(1.5)

Out of those patients who underwent successful pericardiocentesis, 25 (38.4%) required pericardiac window due to recurrent pericardial effusion caused by malignancy. None of the patients died due to the procedure or procedure-related complications.

## Discussion

This is the largest hospital-based study on pericardiocentesis from Pakistan to the best of our knowledge. This study demonstrates that all patients had tamponade physiology at the time of pericardiocentesis, malignancy predominantly lymphoma is the most common cause of pericardial effusion, dyspnea is the most common symptom of cardiac tamponade, and echocardiogram is the most commonly used imaging modality for pericardiocentesis and procedure success is very high with low peri-procedure complications.

Malignancies, predominantly lymphomas, are the most common cause of pericardial effusion in our study population followed by lung carcinoma. This finding is consistent with local and international data. In the study by Khan et al., malignancy was the cause of pericardial effusion in more than 50% of the patients [6]. In another study from Saudi Arabia, in 48.7% of the patients, malignancy was the cause of the pericardial effusion [7]. Malignancies and uremia were reported to be the most common cause in the western population [8,9], while Turank et al. reported that the most common cause of pericardial effusion is idiopathic [10]. The mean age of our study population was 48.9 years as compared to 52.1 years in the study from Saudi Arabia [7].

Dyspnea was the most common presenting symptom in our study population followed by hypotension. In the study by Khan et al., 89% of the patients with pericardial effusion presented with dyspnea [6]. Sagristà-Sauleda et al. reported dyspnea as a presenting symptom in 83% of the patients followed by pleuritic chest pain in 42% of the patients [11]. The presence of inflammatory signs or symptoms, e.g. fever and pleuritic chest, may help to differentiate the different causes of pericardial effusion. Agner et al. observed that absence of inflammatory symptoms, large pericardial effusion, cardiomegaly, and pleural effusion was associated with malignant or tuberculous pericardial effusion [12]. In our study population, the majority of patients had large pericardial effusion and all of them had echocardiographic evidence of cardiac tamponade.

In the majority of the patients, pericardiocentesis was performed under echocardiographic guidance and less than one-third underwent fluoroscopy-guided pericardiocentesis. Worldwide, echocardiography is the most commonly use imaging modality for pericardiocentesis and it is associated with high success rate and low peri-procedure complication [13].

Our study reports a pericardiocentesis success rate of 98.5% with relatively low complications. However, more than one-third of the patients required a pericardial window due to recurrent pericardial effusion. These findings are consistent with the literature. Khan et al. reported a success rate of 95.5% for echocardiography guided pericardiocentesis [6]. Albugami et al. reported re-accumulation of pericardial effusion as high as 21% [7]. Rate of major complications including pneumothorax and myocardial perforation under echocardiographic or fluoroscopy-guided pericardiocentesis is reported to be 0.3 to 3.9% in observational studies [13,14].

Pericardiocentesis was done via a subcostal or subxiphoid approach in our study population, which is reported to be associated with a low risk of pneumothorax [15].

Our study is not free of limitations. It is a retrospective observational study and sample size is small which may not represent the whole Pakistani population. There is no control group, so it is not possible to establish the association of pericardial effusion with mortality. There is no long-term follow-up.

## Conclusions

Malignancy, predominantly lymphoma, is the most common cause of pericardial effusion requiring pericardiocentesis. Dyspnea is the most common symptom of presentation with cardiac tamponade. Echocardiography is the commonly used imaging modality for pericardiocentesis. Bedside setting is the most common setting used for pericardiocentesis. Imaging guided pericardiocentesis has a very high success and low complication rate.

## References

[1] Loukas M, Walters A, Boon JM (2012). Pericardiocentesis: a clinical anatomy review. Clin Anat.

[2] Nguyen CT, Lee E, Luo H (2011). Echocardiographic guidance for diagnostic and therapeutic percutaneous procedures. Cardiovasc Diagn Ther.

[3] Ainsworth CD, Salehian O (2011). Echo-guided pericardiocentesis: let the bubbles show the way. Circulation.

[4] Maggiolini S, Bozzano A, Russo P (2001). Echocardiography-guided pericardiocentesis with probe-mounted needle. J Am Soc Echocardiogr.

[5] Inglis R, King AJ, Gleave M (2011). Pericardiocentesis in contemporary practice. J Invasive Cardiol.

[6] Khan AA, Kazmi KA, Najaf SM (2005). Clinical and echocardiographic characteristics of patients with significant pericardial effusion requiring pericardiocentesis. J Pak Med Assoc.

[7] Albugami S, Al-Husayni F, AlMalki A (2020). Etiology of pericardial effusion and outcomes post pericardiocentesis in the Western region of Saudi Arabia: a single-center experience. Cureus.

[8] Osranek M, Bursi F, O’Leary PW (2003). Hand-carried ultrasound-guided pericardiocentesis and thoracentesis. J Am Soc Echocardiogr.

[9] Strobbe A, Adriaenssens T, Bennett J (2017). Etiology and long‐term outcome of patients undergoing pericardiocentesis. J Am Heart Assoc.

[10] Basar N, Turak O, Gürel M (2012). Pericardial effusion: etiology, diagnose and management. Düzce Med J.

[11] Sagristà-Sauleda J, Mercé AS, Soler-Soler J (2011). Diagnosis and management of pericardial effusion. World J Cardiol.

[12] Agner RC, Gallis HA (1979). Pericarditis: differential diagnostic considerations. Arch Intern Med.

[13] Maggiolini S, Gentile G, Farina A (2016). Safety, efficacy, and complications of pericardiocentesis by the real-time echo-monitored procedure. Am J Cardiol.

[14] Akyuz S, Zengin A, Arugaslan E (2015). Echo-guided pericardiocentesis in patients with clinically significant pericardial effusion. Herz.

[15] De Carlini CC, Maggiolini S (2017). Pericardiocentesis in cardiac tamponade: indications and practical aspect. E-J Cardiol Pract.

